# Kress & Owen *vs.* Crittenden

**Published:** 1905-02

**Authors:** 


### KRESS & OWEN VS. CRITTENDEN.

On the 8th day of December, Police Magistrate Denison, in the police court, registered a conviction against Thomas Crittenden, Jr., who keeps two drug stores in Toronto, one at the corner of Howard and Sherbourne streets, and the other at the corner of Gerrard and Sumach streets, for infringement of the trade mark, duly registered in Canada, owned by Kress & Owen Co., 210 Fulton street, New York, "Glyco-Thymoline." The evidence conclusively showed that the defendant had put up a preparation under the name of "Glyco-Thymol," in bottles almost identical to those of Kress & Owen Co., and with labels worded *verbatim et literatim* to those of the original manufacturers. The magistrate in registering the conviction, gave the defendant's solicitor, who hinted at an appeal, to understand that if he entertained that idea, he would not only fine, but imprison his client as the law provide.d The case was adjourned for a week, at the end of which time Crittenden, through his solicitor, gave an undertaking that he would stop all manufacture of Glyco-Thymol and destroy all labels, bottles, etc., connected with the sale of that preparation. The firm of Kress & Owen Co. are deserving of congratulation over the result of this case. They had every reason for prosecuting Crittenden, as it was nothing short of dishonesty and contrary to law that he should stoop to such practices and try to rob a firm who, by strictly ethical advertising (solely to the profession) and the expenditure of about $175,000 per annum, have secured a large sale of Glyco-Thymoline, a preparation found valuable in catarrhal conditions of the mucous membrane.—*Canadian Journal of Medicine and Surgery, editorial, January, 1905.*

